# Acoustic Complexity of vocal fish communities: a field and controlled validation

**DOI:** 10.1038/s41598-018-28771-6

**Published:** 2018-07-12

**Authors:** Marta Bolgan, M. Clara P. Amorim, Paulo J. Fonseca, Lucia Di Iorio, Eric Parmentier

**Affiliations:** 1 Laboratoire de Morphologie Fonctionnelle et Evolutive, Institut de Chimie—B6C, Université de Liège, Liège, Belgium; 2 MARE, Marine and Environmental Sciences Centre, ISPA - Instituto Universitário, Lisbon, Portugal; 3 Departamento de Biologia Animal and Centre for Ecology, Evolution and Environmental Changes, Faculdade de Ciências, Universidade de Lisboa, Lisbon, Portugal; 4 CHORUS Institute, INP Phelma Minatec, 3 Parvis Louis Néel, 38016 Grenoble, France

## Abstract

The Acoustic Complexity Index (ACI) is increasingly applied to the study of biodiversity in aquatic habitats. However, it remains unknown which types of acoustic information are highlighted by this index in underwater environments. This study explored the robustness of the ACI to fine variations in fish sound abundance (i.e. number of sounds) and sound diversity (i.e. number of sound types) in field recordings and controlled experiments. The ACI was found to be sensitive to variations in both sound abundance and sound diversity, making it difficult to discern between these variables. Furthermore, the ACI was strongly dependent on the settings used for its calculation (i.e. frequency and temporal resolution of the ACI algorithm, amplitude filter). Care should thus be taken when comparing ACI absolute values between studies, or between sites with site-specific characteristics (e.g. species diversity, fish vocal community composition). As the use of ecoacoustic indices presents a promising tool for the monitoring of vulnerable environments, methodological validations like those presented in this paper are of paramount importance in understanding which biologically important information can be gathered by applying acoustic indices to Passive Acoustic Monitoring data.

## Introduction

Since the mid-20th century, human-induced ecosystem changes have been occurring with increased rapidity^1^. In this context of global-scale, long-term changes, it is necessary to undertake long-term monitoring of both habitats and wildlife, adopting an ecosystem-based approach. Recently, Krause and Farina^2^ have suggested the use of Passive Acoustic Monitoring (PAM) for understanding long-term changes in community richness and diversity. In aquatic environments, PAM involves the use of hydrophones to record all components of underwater soundscapes, including environmental noise, animal communicative and involuntary sounds, as well as anthropogenic noise. Although PAM has been primarily applied in relation to marine mammals, it is now used to investigate several aspects of vocal fish populations, such as presence, distribution, diel cycle of activity, social interactions, seasonal movements, as well as for delimiting spawning seasons and areas^3–9^. Despite this, Tricas & Boyle^10^ and Ruppé *et al*.^11^ have pointed out a general paucity of data addressing the acoustic communication of fish living in natural communities. This lack of knowledge probably results from the fact that acoustic data have traditionally been analysed through the manual and aural quantification of sound occurrences, which is an extremely time-consuming procedure. Two automated processing methods allow for a significant reduction in the time required to process large acoustic datasets: (i) approaches based on the automatic detection of calls, including Gaussian mixture models, artificial neural networks, or hidden Markov models among others^12^, or (ii) the application of acoustic indices^13^, such as acoustic richness (AR^14^), temporal and frequency entropies (H_t_ and H_f_^15^), the Acoustic Diversity Index (ADI^16^) and the Acoustic Complexity Index (ACI^17,18^). Both methods have primarily been applied to the vocalisations of insects, amphibians, birds, and mammals occurring in terrestrial soundscapes. On the other hand, developments in the automatic detection of fish calls are still in their infancy, although some recent studies are worth mentioning^12,19–21^. One of the reasons for the current lack of automatic fish call detectors is that the development of certain automatic detection methods (e.g. Markov models) requires *a priori* knowledge of the recorded sounds. This can be problematic, as the acoustic repertoire of many fish species remains to be described. On the other hand, the use of acoustic indices to unravel complex biophonic patterns does not require prior knowledge of the targeted signals (at least theoretically), and is of straightforward application.

**Figure 1:**
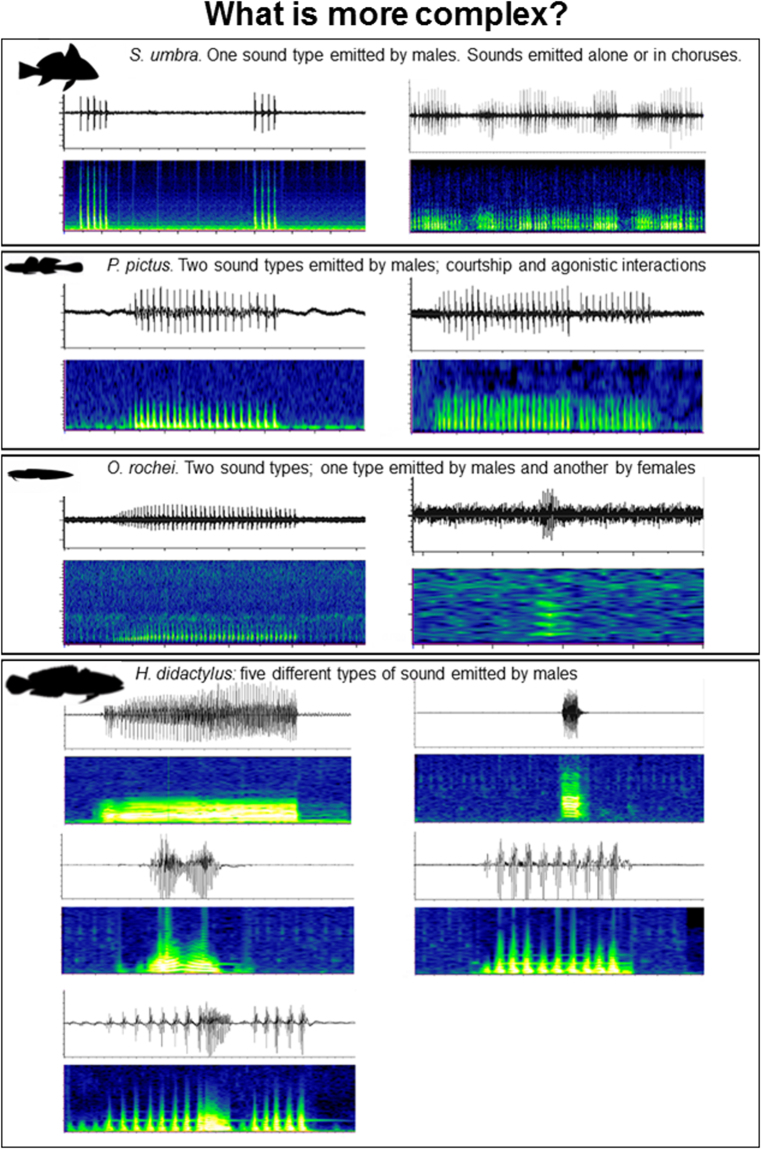
The acoustic complexity of vocal fish communities (measured at a specific moment in space and time) can be function of (i) variations of a single species call rate (e.g. brown meagre, *Sciaena umbra*), (ii) the number of different sound types, and (iii) the relative abundance of each sound type. In fish vocal communities, the number of sound types might not correspond to the number of vocal species or individuals present on site. The number of sound types can be a function of behavioural, sexual, ontogenetic, and physiological differences within the same species (e.g. the painted goby *Pomatoschistus pictus*, the Roche’s snake blenny *Ophidion rochei*, and the Lusitanian toadfish *Halobatrachus didactylus*, a species characterised by high acoustic disparity).

Aquatic soundscapes differ from terrestrial ones, being characterised by fewer frequency-modulated biological sound sources (cetacean sounds being a prominent exception). Additionally, a narrow low-frequency band is mostly occupied by temporally patterned fish vocalisations, and very frequent unpatterned broadband sounds originating from benthic organisms are superimposed to different levels of environmental noise. Despite these differences, an increasing number of studies have applied the ACI to aquatic environments in order to gain information about diversity or ecological state^22–32^. This index measures the complexity of the biophonical component, providing an indirect and immediate measure of biogenic sound dynamics^18^. The ACI applied to avian vocal communities correlates with the number of bird songs by highlighting rapid variations of intensity in each single frequency bin, a feature which is typical of bird vocalizations^18^. However, to date, no study has investigated in detail which type of acoustic information is highlighted by the ACI in aquatic habitats. Furthermore, for vocal fish communities, the definition of acoustic complexity is not always straightforward, i.e. what does ‘complexity’ actually mean, and does sound complexity reflect species diversity? In the modern Oxford dictionary, ‘complex’ is defined as “made of several closely connected parts”. Therefore, there are two main dimensions characterising complexity: connection (i.e. dependency) and distinction (i.e. heterogeneity, diversity and disparity)^33^. The search for global patterns of biodiversity mobilises a range of measures^34^. Species richness – based on the presence or absence of different species – is one facet of diversity, but a thorough characterisation must account for the variety of species as well as for their abundance. Complexity of fish vocal communities is not only difficult to define but also to quantify. What can be considered more complex? An environment in which many fish species vocalise (i.e. high sound diversity), an environment in which few species consistently increase their vocalisation rate (i.e. high abundance of sounds), or an acoustic environment filled by sounds of few species with a variable acoustic repertoire (high acoustic disparity) (see Fig. 1)? Or, indeed, is an environment in which many individuals vocalise, but at variable distances from the hydrophone, more complex?

Within the scope of habitat monitoring and management, a detailed understanding of how acoustic indices discern between diversity, abundance, and the interaction between the two is of fundamental importance. Rice *et al*.^29^ stated that one of the principal challenges in using acoustic indices to explore large data sets is the ability to discern how specific acoustic events contribute to a particular index. Furthermore, it is unclear how differences in sampling rate and spectral resolution impact the performance of an index. In particular, regarding the ACI, it remains unknown if this index highlights the abundance of sounds or the number of sound types (sound diversity and/or disparity), and if it is possible to discern between these two types of information. As the ACI calculates the absolute difference (d_k_) between two adjacent values of intensity (I_k_ and I_(k+1)_) in a single frequency bin (∆_fi_), and then adds together all of the d_k_ encompassed in the recording analysis window, our hypothesis was that the ACI is sensitive to variations in both sound abundance and sound diversity, but may not discriminate between the two.

The aim of this study was therefore to verify which types of acoustic information are provided by the ACI, and to test the effects of the settings (i.e. spectral and temporal resolution of the ACI algorithm, amplitude filter) on the ACI output. The ACI was evaluated in a controlled experiment and in natural conditions (field recordings; wild fish communities).

## Results

### The Effect of Operator Choice on the ACI

#### Spectral resolution ∆fi

The ACI was found to be strongly influenced by all settings that must be chosen prior to its calculation.

**Figure 2:**
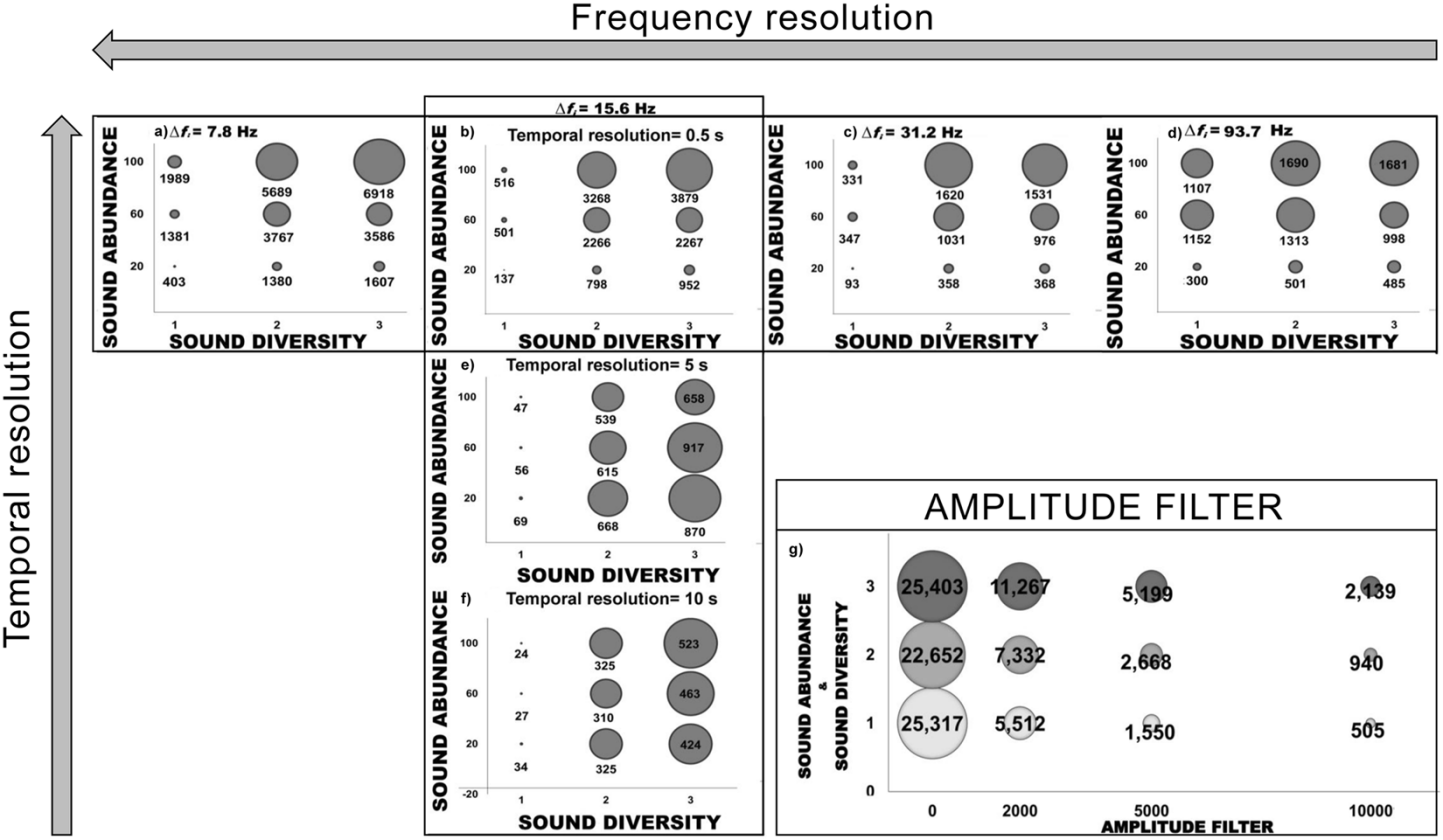
The effect of the settings on the ACI, where the size of the bubbles and their labels represent the value of the ACIsum (controlled experiment CE). (**a**–**d**) Show the influence of the frequency resolution (∆*f*_*i*,_ on CE_1_); (**b**,**e** and **f**) show the influence of the temporal resolution of the ACI algorithm (CE_1_); and (**g**) shows the effect of the amplitude filter (μV^2^/Hz) in the presence of fish choruses (CE_2_), where (1) is the chorus of *S*. *umbra*, (2) is the chorus of *S*. *umbra* with 20 sounds ∙ min^−1^ of *O*. *rochei* and 20 *Kwa* sounds ∙ min^−1^, and (3) is the chorus of *S*. *umbra* with 30 sounds ∙ min^−1^ of *O*. *rochei* and 30 *Kwa* sounds ∙ min^−1^.

In our controlled experiment (CE_1_), the ACI was significantly influenced by the choice of ∆f_i_ (Fig. 2a–d and Supplementary Table 1). Furthermore, the ACI calculated using ∆f_i_ = 31.2 Hz and ∆f_i_ = 93.7 Hz did not correctly represent abundance variations in sounds of the same type (higher abundance of one sound type corresponded to lower ACI values). In particular, the ACI calculated using ∆f_i_ = 93.7 Hz was not correlated with variations in sound abundance (∆f_i_ = 93.7, r_s_ = 0.52, p-value = 0.144). Sound diversity variations were not correctly represented by ∆f_i_ = 7.8 Hz and ∆f_i_ = 31.2 Hz at mid-range abundance (i.e. 60 sounds ∙ min^−1^, Fig. 2a–c), and by the ACI calculated using ∆f_i_ = 31.2 Hz at high abundance (i.e. 100 sounds ∙ min^−1^, Fig. 2c). Furthermore, the ACI calculated using ∆f_i_ = 93.7 Hz never correctly represented variations in sound diversity (Fig. 2d). The ACI calculated with ∆f_i_ = 7.8 Hz and with ∆f_i_ = 93.7 Hz were not correlated with variations in sound diversity (∆f_i_ = 7.8 Hz, r_s_ = 0.52, p-value = 0.144, ∆f_i_ = 93.7, r_s_ = 0.10, p-value = 0.787). In the analysis of the field recordings (F), a similar influence of the chosen ∆f_i_ on the ACI was found. Indeed, the ACI differed significantly when calculated using different frequency resolutions (∆f_i 1_ = 15.6 Hz, ∆f_i 2_ = 93.75 Hz; U_(613,613)_ = 9194, p-value = 0.001).

#### Temporal resolution of the ACI algorithm

In the analysis of the controlled experiment (CE_1_), different temporal resolution of the ACI algorithm resulted in similar patterns of the ACI, but in significantly different ACI values (Fig. 2b–f and Supplementary Table 1). Sound abundance was not correctly represented by temporal resolutions of 5 s (r_s_ = −0.36, p-value = 0.328) (Fig. 2e) or 10 s (r_s_ = −0.05, p-value = 0.890) (Fig. 2f); lower abundance of sounds resulted in higher ACI values, and there was no correlation between the ACI and sound abundance.

#### Amplitude filter (noise filter)

**Figure 3:**
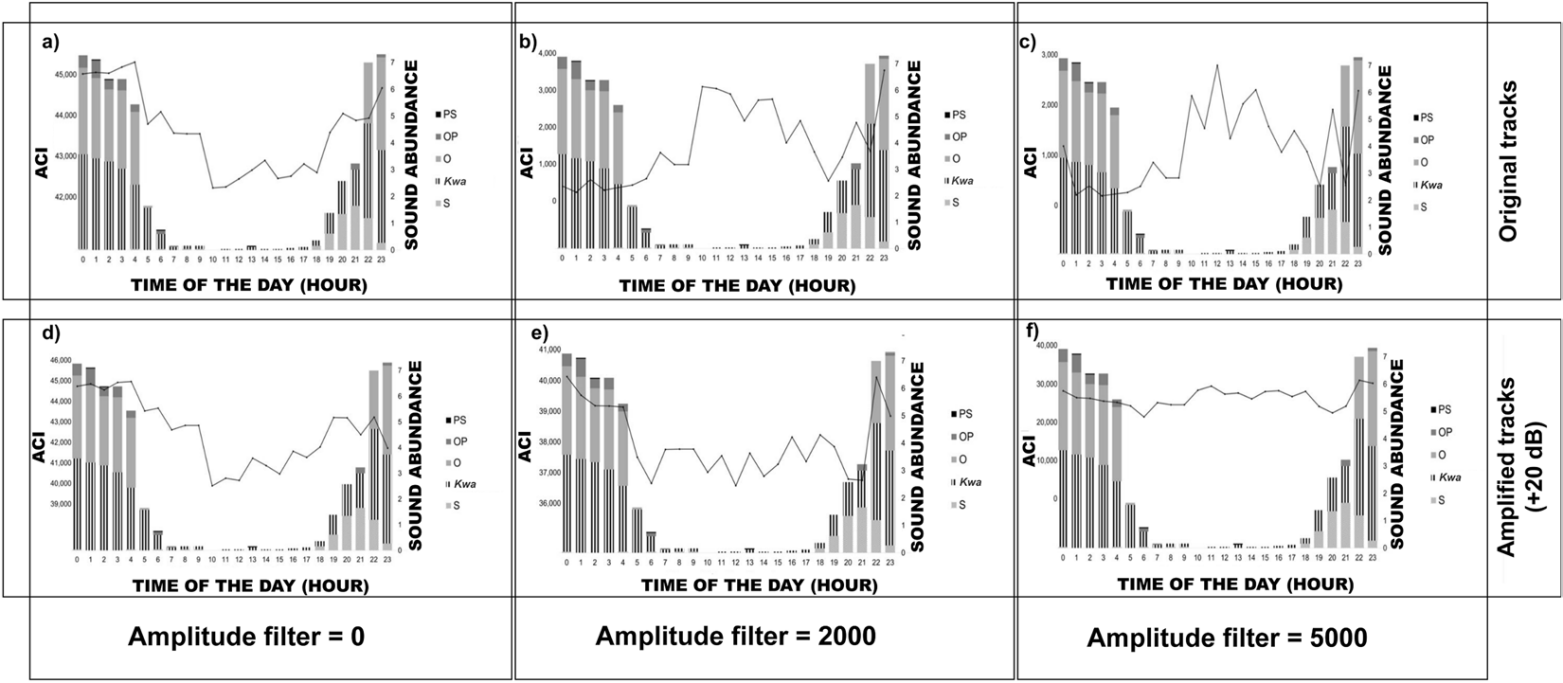
The influence of the amplitude filter and of recording characteristics (i.e. gain) on the ACI (ACIsum, Calvi, France, field recordings F). Abundance of different sound types (histogram bars) and ACI (black line) in the wild fish vocal community over a 24 h cycle. S = *Sciaenidae* sounds; O = *O*. *rochei* sounds (male); *Kwa* = harmonic sounds of unknown origin; PS = pulse series sounds of unknown origin and OP = single pulse sounds of unknown origin. (**a**) ACI calculated on unamplified tracks with amplitude filter deactivated; (**b**) ACI calculated on unamplified tracks with 2000 μV^2^/Hz amplitude filter; (**c**) ACI calculated on unamplified tracks with 5000 μV^2^/Hz amplitude filter; (**d**) ACI calculated on amplified tracks (+20 dB) with amplitude filter deactivated; (**e**) ACI calculated on amplified tracks (+20 dB) with 2000 μV^2^/Hz amplitude filter; (**f**) ACI calculated on amplified tracks (+20 dB) with 5000 μV^2^/Hz amplitude filter.

The ACI was significantly influenced by the choice of amplitude filter (Fig. 2g and Supplementary Table 2). In our controlled experiment (CE_2_), the ACI was not sensitive to abundance variation in *O*. *rochei* and *Kwa* sounds in presence of *S*. *umbra* chorus when the amplitude filter was not applied (Fig. 2g). The application of the amplitude filter resulted in a better discrimination of abundance variation in *O*. *rochei* and *Kwa* sounds (Fig. 2g). The application of different amplitude filters gave statistically different ACI values when applied to the field recordings (H_(5,3678)_ = 3295, p-value = 0.001). In the field recordings (F), the best representation of the vocal fish community occurred when the filter was not applied (highest correlation coefficients, Supplementary Table 3 and Fig. 3a/d). When the filter was applied (5000 μV^2^/Hz or 2000 μV^2^/Hz Fig. 3b–f), the ACI peaked during daytime hours (i.e. when anthropogenic noise was enhanced by boat traffic) and fell when fish vocalisations were most abundant and diverse (night-time hours; noise filter = 5000 μV^2^/Hz; Fig. 3b,c,f). However, when a 2000 μV^2^/Hz filter was applied to tracks amplified by 20 dB, the ACI peaked at night-time hours, concurrent with the highest number and diversity of fish sounds (Fig. 3e).

The ACI therefore appears to be strongly influenced by all settings chosen prior to its calculation. Furthermore, it appears that each specific situation requires *ad hoc* settings for the ACI to be representative of variation in fish sound abundance and diversity. In our controlled experiment (CE), the parameters that provided a better representation of sound abundance and sound diversity were ∆f_i_ = 15.6 Hz (sampling rate 8 kHz, FFT 512 Hanning), temporal resolution = 0.5 s and amplitude filter = 5000 μV^2^/Hz. In our field recordings (F), on the other hand, a better representation was found with similar frequency and temporal resolution, but with the amplitude filter deactivated. The interpretation of the information provided by the ACI was therefore carried out by using these specific settings for each of the two situations (CE and F).

### Information provided by the ACI

**Figure 4:**
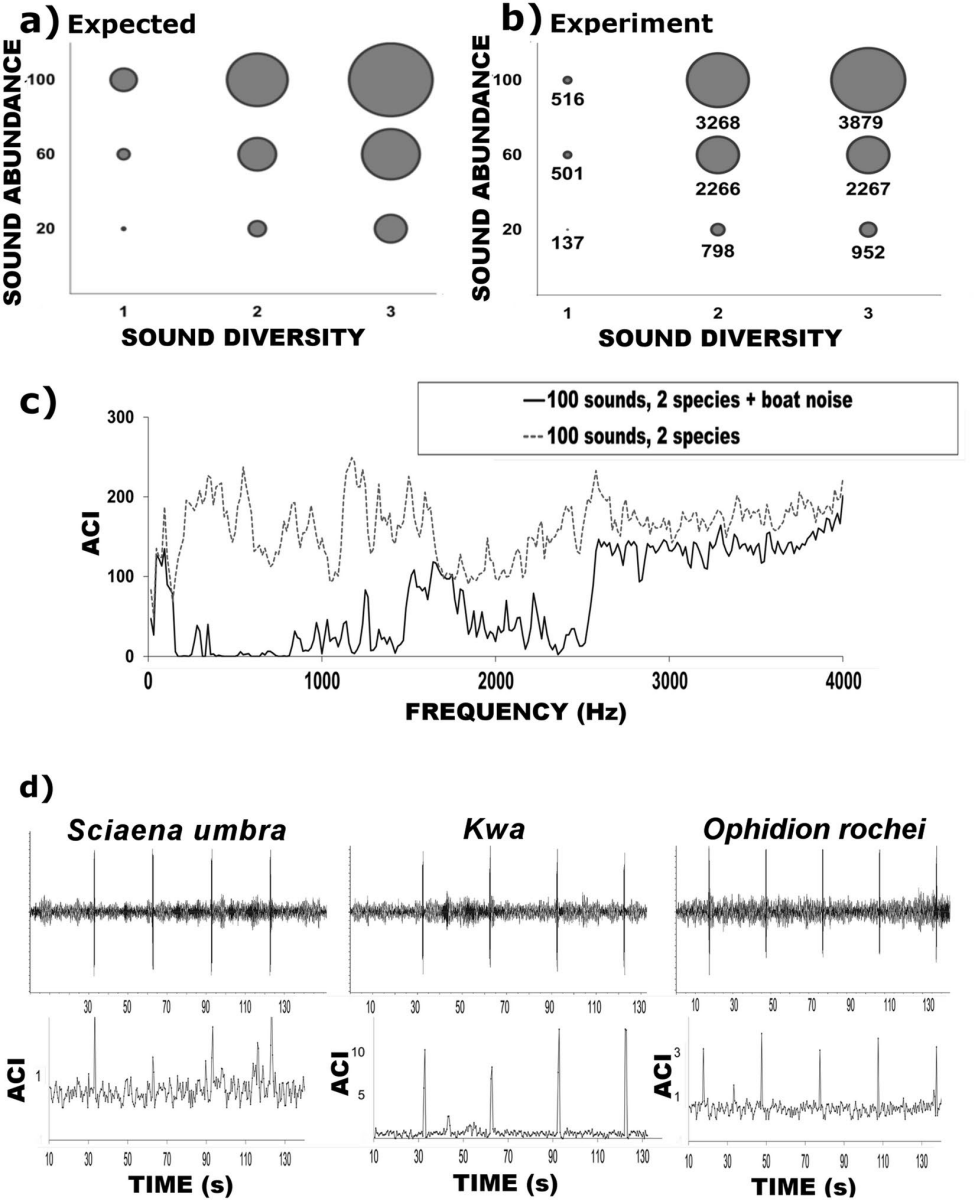
ACI behaviour during the controlled experiment (CE). (**a**,**b**) Depict ACI behaviour (ACIsum) in the presence of controlled variations in sound abundance and/or of sound diversity (CE_1_); (**c**) depicts the behaviour of the ACI in presence of boat noise (ACI values plotted over frequency, CE_3_); and (**d**) depicts the behaviour of the ACI in the presence of rare fish sound events (2 sounds ∙ min^−1^, CE_4_). The waveform of the playback recording is shown on top, with the corresponding ACI values plotted over the same time-scale on the bottom graphs.

In the controlled experiment (CE_1_), higher values of ACI corresponded to more numerous sounds and/or more numerous sound types (Fig. 4a,b). Statistically, the ACI was positively correlated with both sound abundance (r_s_ = 0.66; p-value = 0.03) and sound diversity (r_s_ = 0.85; p-value = 0.001). Furthermore, the ACI was found to be sensitive to the presence of boat noise (CE_3_): when the same biophony (i.e. 100 sounds ∙ min^−1^ of *S*. *umbra* and *Kwa* sounds) was played back alongside boat noise, the ACI decreased significantly (Fig. 4c, U = 67573, p-value = 0.001). The presence of boat noise induced a significant decrease of the ACI, regardless of the type or composition of the biophony, i.e. 100 sounds ∙ min^−1^ of *S*. *umbra* and of *Kwa* sounds (U = 67573, p-value = 0.001), 60 sounds ∙ min^−1^ of *S*. *umbra* and *Kwa* sounds (U = 93230, p-value = 0.01), or 20 sounds ∙ min^−1^ of *O*. *rochei* (U = 88348, p-value = 0.01). In the presence of boat noise, no correlation was found between the ACI and the number of emitted sounds (r_s_ = 0.025; p-value = 0.447).

In conditions absent of anthrophony, the ACI appeared sensitive to the presence of rare biophonical events, i.e. 2 sounds ∙ min^−1^ of *S*. *umbra*, *O*. *rochei* and *Kwa* sounds, when plotted over time (CE_4_, Fig. 4d). However, the type of biophonical event provided different results (i.e. type of sounds, H_(3,1020)_ = 117.05; p-value = 0.001). In particular, there was a significant difference between the ACI calculated for the pool’s background noise and the one calculated when two *Kwa* sounds ∙ min^−1^ were played back (U = 23938; p-value = 0.001), but no statistically significant difference was found in the case of *S*. *umbra* (U = 32479; p-value = 0.950) and of *O*. *rochei* (U = 31348; p-value = 0.480).

**Figure 5:**
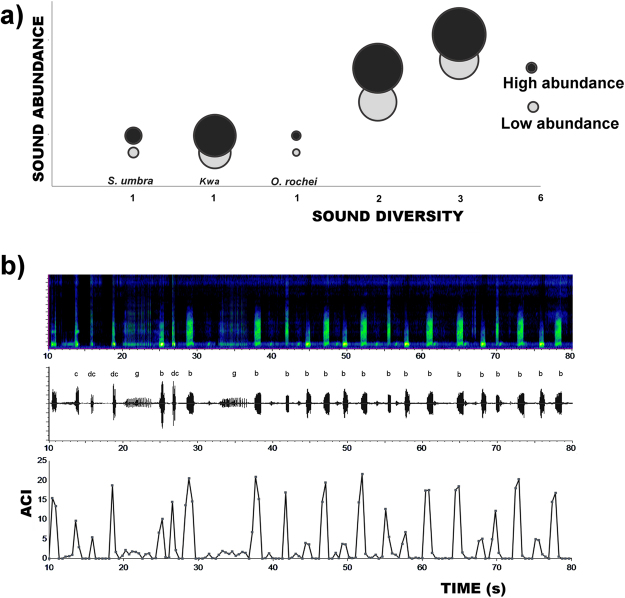
ACI behaviour during the controlled experiment (CE). (**a**) ACIsum of fish sounds at their real duration (CE_5_); (**b**) ACI values over time during the playback of sounds of *H*. *didactylus* (one species emitting multiple sound types, CE_6_). Spectrogram and waveform of the playback recording is shown on top (Hanning, FFT 512, 50% overlap), the corresponding ACI values are plotted over the same time-scale on the bottom. c = croak; dc = double croak; g = grunt; b = boatwhistle.

When fish sounds were played back at their real duration (CE_5_), the ACI was found to positively correlate, with similar strength, to both sound abundance (rs = 0.42; p-value = 0.001) and sound diversity (rs = 0.44; p-value = 0.001, Fig. 5a). When comparing the ACI calculated in the presence of fifty sounds ∙ min^−1^ of *S*. *umbra* with the ACI calculated in the presence of fifty *Kwa* sounds ∙ min^−1^, a significant difference was found (U = 24125; p-value = 0.001), suggesting that the ACI has a different sensitivity to distinct types of sounds. This differential sensitivity to different types of biophonical events was confirmed during the playback of different sound types from the same species (CE_6_, *H*. *didactylus*, H_(5,41)_ = 2608, p-value = 0.001, Fig. 5b). For example, the ACI of a boatwhistle was higher (ACI mean = 10.73) than a grunt train in *H*. *didactylus* (ACI = 7.0).

**Figure 6:**
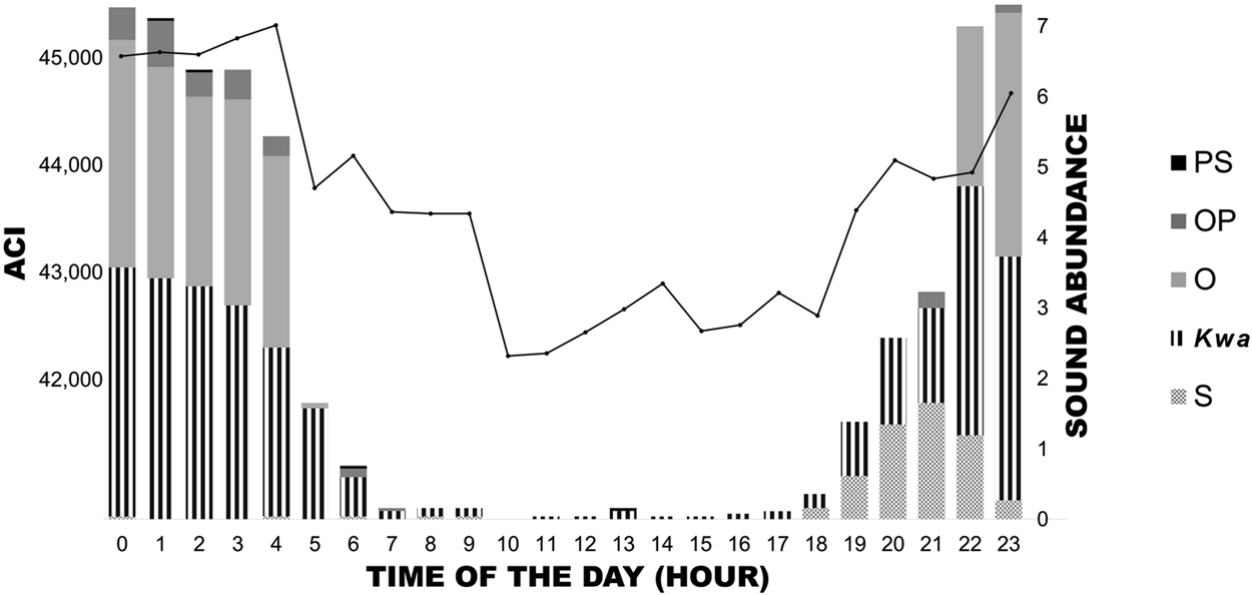
The fish vocal community at 40 m depth (sandy bottom) in Calvi (France) over a 24 h cycle (June). Abundance of different sound types (histogram bars) and ACI correspondence (black line, ACIsum). S = *Sciaenidae* sounds; O = *O*. *rochei* sounds (male); *Kwa* = harmonic sounds of unknown origin; PS = pulse series sounds of unknown origin; and OP = single pulse sounds of unknown origin.

In the field recordings (F), the results of manual scrolling show that the fish vocal community recorded at a depth of 40 m (sandy-bottom, inferior margin of *Posidonia oceanica* meadows) in Calvi (France) during June 2013 is characterised by diel variation, with the highest call rate occurring at night (Fig. 6). Although higher values of ACI (hourly data) did not always correspond to higher scores of abundance/diversity of sounds from manual selections (Fig. 6, e.g. 4 am), the overall pattern of the ACI followed the pattern of fish sounds and was positively correlated with both sound abundance and sound diversity (Supplementary Table 4).

## Discussion

Ecoacoustics is a powerful new method for the long-term monitoring of population ecologies and dynamics^2^. Acoustic indices, originally developed for terrestrial soundscapes, are increasingly applied to describe biodiversity or the ecological state of aquatic habitats. Despite of this, no study to date has investigated in details which type of acoustic information (i.e. sound abundance and/or sound diversity) is highlighted by the ACI when applied to specific marine vocal communities. In this study, the ACI was found to be strongly influenced by all settings, i.e. frequency and temporal resolution of the ACI algorithm, and the amplitude filter. These results imply a high flexibility of the ACI to different situations but also that users must have a good knowledge of the implications given by the choice of the ACI settings. Moreover, care has to be taken when using this index to compare different studies, or sites with site-specific characteristics (e.g. species diversity, quality indices), as the ACI absolute values should not be considered alone. This is especially important if different soundscapes are compared and different settings for calculating the ACI are used.

Our results regarding the influence of the amplitude filter on ACI efficiency are in accordance with^32^. In our controlled experiment, the amplitude filter was indeed required to discriminate small variations of *O*. *rochei* and *Kwa* sounds in the presence of *S*. *umbra* chorus. However, when the amplitude filter was applied to our field recordings in Corsica (F), the ACI highlighted boat noise rather than fish vocalisation. These results could be explained by the fact that fish sounds are less intense than anthropogenic noise, and were therefore cut off by the amplitude filter due to their low signal-to-noise ratio. The authors of^32^ concluded that the use of the filter reduced the power of attenuation of boat noise in the frequency band shared with fish sounds. Our field recordings were obtained in similar acoustic environments (i.e. *P*. *oceanica* meadows), and the ACI was calculated using similar frequency and temporal resolution. However, our results regarding the efficiency of the amplitude filter differ (i.e. the application of the noise filter resulted in an inversed pattern of the ACI, see Fig. 5). We therefore suggest that the ACI may be dependent not only on adjustments of the algorithm settings to the biophony being analysed, but may also be affected by the characteristics of the recording chain used to collect the data, such as the gain (see Fig. 3e). At the light of this suggestion, further investigations regarding the influence of different recording chains and acquisition settings on the ACI output are encouraged.

When the settings providing the best discrimination of fine variations in the biophonical community were identified and applied in this study, the ACI was positively correlated with sound abundance and sound diversity, in both controlled experiments and in the field. This study therefore supports the finding that the ACI *per se* does not provide a sufficient and valuable discrimination between sound abundance and sound diversity. This discrimination is of fundamental importance in describing biodiversity and habitat quality and in studying their dynamics over extended periods of time. The concept of complexity is based on the assumption that a higher number of singing individuals and species will result in an increase in acoustic complexity^13^. Although it is likely that a higher number of vocal species results in a higher abundance of sounds and of sound types, this might not always be the case. For example, a high number of sounds could correspond to low species diversity, if these are emitted by one or few species vocalising at high rates (e.g. brown meagre and *Kwa* sounds, see Fig. 1 and^35^). Such variations in calling rate (i.e. number of calls) are often linked to the season or the time of the day in which recordings are collected (e.g.^8^). The presence of acoustically overwhelming species is very common in soundscapes across different habitats, and this “masks” the effect of acoustic diversity on the ACI^35^.

In general terms, care should be taken when linking high sound diversity to high species diversity. It is possible that a high level of sound diversity corresponds to a low level of species diversity; for example, in the presence of species characterised by high acoustic disparity, such as the Lusitanian toadfish, which produces five different sound types (see Fig. 1). The application of acoustic indices alone, without a prior knowledge of the type of signals present in a specific site, might result in interpretations that do not accurately reflect the biodiversity and the ecological status of the area. We conclude that the application of validation procedures, such as the one presented in this study (i.e. controlled experiments and/or comparison with a subsample of the data on which manual scrolling has been carried out), can improve the resolution and the quality of the information extrapolated by acoustic indices.

In particular, by manually scrolling a subsample of the data, the robustness of the index to the specific acoustic soundscape being analysed can be investigated, and the settings providing the best possible resolution of sound events variation can be identified. Finally, manual scrolling has the potential to provide additional biologically important information, where the application of acoustic indices alone will result in losing this information. For example, Montie *et al*.^36^ and Henry *et al*.^37^ have shown that spawning red drum *Sciaenops ocellatus* emit sounds of longer durations and with more pulses than non-spawning individuals, demonstrating that acoustic metrics can provide quantitative information of spawning in the wild. Monitoring wild fish spawning can be critical for understanding factors relating to changes in population abundance, and therefore to the management of endangered or commercially important species. If this information can be gathered through a completely non-invasive technique such as PAM, the ability to discern between spawning and non-spawning individuals still presently relies on the operator’s manual analysis (but see^12^).

The development of new tools is recommended, tailored to fish sounds, allowing the automatic analysis of large acoustic datasets, and providing a higher resolution and discrimination in terms of sound abundance and sound diversity dynamics. The temporal patterns of fish calls (spanning from their rhythms and calling rates, to their duration, number of pulses, and pulse period), contain diverse useful information. For example, the progression of the pulse period within a call can help distinguish the species emitting the calls^38^, and can be indicative of the mechanisms of sound production^39,40^. Calling rate and sound duration can convey information about male status and spawning readiness to females^36,37^; calling rate and pulse period can be related to male body condition^40^; and an increase in pulse number (and pulse rate) can be motivation-dependent, probably carrying multiple messages regarding the sender’s quality, including size, condition, and motivation^41^. Furthermore, the temporal patterns of fish sounds are less affected by propagation losses or distortion than spectral or amplitude features^42^. The development of novel approaches for describing long-term patterns of fish populations and their dynamics should therefore focus on the temporal features of their calls.

In conclusion, the ACI has the potential to quickly unravel biophonic patterns, especially when long-term studies are undertaken from an ecoacoustic perspective. The ACI, if used carefully and together with other methods for assessing the state of the underwater ecosystem, may be useful tools for assessing variations when comparing the same site along time, as it may allow to quickly spot changes, calling for more detailed investigations. However, it has to be reminded that, when the ACI is applied to fish vocal communities, it does not discriminate between sound abundance and sound diversity. Finally, care should be taken when comparing ACI absolute values between studies as these are strongly dependent on its settings.

## Methods

### Controlled experiment

The controlled experiments (CE) were designed to test the influence of sound abundance and sound diversity on the ACI. We created artificial tracks that were played back in a large pool using loudspeakers. The output of this playback was recorded via a hydrophone. The ACI was calculated on the tracks recorded via this hydrophone. Spearman rank correlations were used to investigate the strength of the relationship between the calculated ACI and the number of sounds (i.e. sound abundance), and/or number of sound types (sound diversity).

#### Sound stimuli and experimental design

For the creation of the artificial tracks used for the CE, three types of fish sounds were selected (Supplementary Fig. 1a): (i) the *Sciaena umbra* (peak freq. 250 Hz, 1^st^ quartile freq. 187 Hz, 3^rd^ quartile freq. 406 Hz, recorded in the Adriatic Sea, Italy^7^), (ii) the “Kwa” (peak freq. 781 Hz, 1^st^ quartile freq. 681 Hz, 3^rd^ quartile freq. 1187 Hz, unidentified fish sounds from Mediterranean *Posidonia oceanica* meadows; sound recorded in Sardinia, Italy^35^), and (iii) *Ophidion rochei* (peak freq. 281 Hz, 1^st^ quartile freq. 156 Hz, 3^rd^ quartile freq. 312 Hz, recorded by L. Kéver in Calvi, France). Using Adobe Audition, these sounds were amplitude equalised to avoid bias linked to amplitude differences between them (Supplementary Fig. 1b, waveform).

To test the effect of sound abundance on the ACI (independently of the type of sound used), the durations of different call types had to be standardised. Because each sound type is characterised by a specific duration, they were cut to the same duration (i.e. 500 ms sound stimuli; Supplementary Fig. 1a). Three-minutes tracks were created, alternating the 500 ms sound stimuli with lower-amplitude white noise (SNR > 48 dB). Each three-minute file included one sound type only (Supplementary Fig. 1b); when different sound types were used, each loudspeaker was used for playing back one sound type. We conducted an additional test (not presented in the results) in which we tested the effect of this choice (i.e. each loudspeaker playing back one species only), where additional artificial tracks were played back by a single loudspeaker. This additional set of tracks was created by including the same number of sounds presented during CE_1_, but with different sound types included in one single track. The ACIsum calculated on these tracks did not differ from the ACIsum calculated during CE_1_ by using different loudspeakers for different sound types (U = 28.5, p-value = 0.309).

Different tracks were used to conduct six experiments, aimed at investigating different aspects of ACI robustness. In particular:

1. CE_1_ = the effect of sound abundance (i.e. number of sounds) and sound diversity (i.e. number of sound types) variations (Supplementary Fig. 1c and Supplementary Table 5);
2. CE_2_ = the effect of a fish chorus (i.e. mass production of sounds of the same type, recorded in the Adriatic Sea, Italy^7^) (Supplementary Table 5);
3. CE_3_ = the effect of boat noise (Supplementary Table 5);
4. CE_4_ = the effect of rare sounds (Supplementary Table 5);
5. CE_5_ = Sound abundance vs. sound diversity, with sounds at their natural duration (Supplementary Table 5);
6. CE_6_ = a case of high acoustic disparity. This experiment consisted of the playback from one loudspeaker (UW-30) of a sound file containing five different sound types emitted by one species only (i.e. *Halobatrachus didactylus*, boatwhistles, grunt trains, croaks, double croaks, and mixed grunt–croak call^43^ see also Fig. 1). This was a blind experiment, as the exact number of sounds of each sound type was unknown to the authors prior to both playback and ACI calculation.

#### Sound stimuli playback and recordings

The sound stimuli playback and recordings were carried out in March 2017, in a tank of 6 m × 9 m × 1.30 m, using four loudspeakers (three UW-30, Electrovoice and one Aqua30, DNH) and two hydrophones (HTI 94 SSQ, sensitivity −162 dB re 1 V/μPa, recording at 48 kHz, and a Brüel & Kjær 8104; sensitivity: −205 dB re 1 V/µPa) placed in the centre of the tank (Supplementary Fig. 1d). The distance from hydrophones to loudspeakers was 90 cm (i.e. within the attenuation distance for a sound at 200 Hz, see^44^).

Each loudspeaker was attached to an amplifier (Blaupunkt GTA 260, Phoenix Gold QX4040 and Sony XM-N1004), that was connected to a USB D-A converter (Edirol U25-EX, 16 bit, 48 kHz) controlled by laptops running Adobe Audition 3.0 (Adobe Systems Inc., Mountain View, CA, USA) that produced the sound stimuli. The Bruel & Kjær 8104 hydrophone, which was connected to a Bruel & Kjær 2238 Mediator Sound Level Meter (Bruel & Kjær, Naerum, Denmark) was used at the beginning of the experiment to equalise the amplitude of all loudspeakers, and to measure the sound pressure level of the background noise and of the signals (93 and 113 dB re 1 μPa, respectively, at 90 cm). The ACI was calculated on the.wav recordings obtained with the HTI 94 SSQ connected to the USB A-D converter (Edirol U25-EX, 48 kHz, 16 bits), and controlled by a laptop through Adobe Audition 3.0.

#### Acoustic Complexity Index calculation and statistical analysis

Because fish sounds occupy a narrow low-frequency band (<2 kHz^45^), each three-minute recording collected by the HTI 94 SSQ during the CE was down-sampled to 8 kHz. These were then processed, in order to calculate the ACI through the open source acoustic program Wavesurfer (v1.8), using a plug-in SoundscapeMeter developed by Farina *et al*.^46^ (Supplementary Table 6). We aimed to investigate (i) the effect of the operator’s choice (i.e. ACI settings) on the ACI, and (ii) the type of acoustic information provided by the ACI when applied to fish sounds.

The settings which must be chosen by the operator in order to calculate the ACI are i) frequency resolution (∆fi = sample rate/FFT segment size), and ii) temporal resolution (s) of the ACI algorithm (Supplementary Table 6). The ACI was computed across the frequency range 0–4000 Hz using an FFT size (Hanning window) of (i) 256 (∆fi = 31.250 Hz), (ii) 512 (∆fi = 15.625 Hz), iii) 1024 (∆fi = 7.812 Hz), and iv) 2048 (∆fi = 3.906 Hz). All these analyses were carried out by setting the temporal resolution of the ACI algorithm at 0.5 s (called clump in the Soundscapemeter manual, see Supplementary Table 6) and the amplitude filter at 5000 μV^2^/Hz. Furthermore, the ACI was re-calculated across the frequency range 0–4000 Hz using a FFT size of 512 (Hanning window) by setting the temporal resolution at 5 s or 10 s. Finally, the ACI was also calculated on the original tracks (48 kHz sampling rate) using an FFT size of 512 (∆fi = 93.750 Hz).

Another setting that can be chosen by the operator when computing the ACI is the amplitude filter (μV^2^/Hz; called “noise filter” in the Soundscapemeter manual, see Supplementary Table 6). The amplitude filter excludes from the computation all data that has an amplitude equal to, or less than, the selected value. The amplitude filter has previously been applied by Buscaino *et al*.^25^ to avoid bias due to highly abundant sounds. We tested its influence on the detection of small variations in fish sound abundance (2 sound types: *O*. *rochei* and *Kwa* sounds) in the presence of another species chorus (i.e. *S*. *umbra*, as recorded in the field^7^).

All ACI values were plotted and visually evaluated to establish the range of maximum variation. ACI values were finally summed (ACIsum, see Supplementary Table 6) for the frequency band 0–2000 Hz (a frequency range encompassing most fish sounds). Non-parametric analysis was carried out using the STATISTICA software. Mann Whitney U and Kruskal H. Wallis tests were used to compare the absolute values of the ACI obtained on the same tracks by changing the frequency and temporal resolution settings as well as the amplitude filter, while Spearman rank correlation was used to investigate the strength of the relationship between the ACI and the number of sounds (i.e. sound abundance) and/or the number of sound types (sound diversity).

### Field recordings

Manual and aural quantification of sound occurrences was carried out on field recordings (F), where both number of sound types (sound diversity) and their abundance (sound abundance) were annotated. The ACI was calculated on the same tracks, and Spearman correlation was used to investigate the strength of the relationship between the ACI and the number of sounds (i.e. sound abundance) and/or the number of sound types (sound diversity).

#### Data collection

A mini-Digital Spectrogram Long-Term Acoustic Recorder (DSG, hydrophone sensitivity; −180 dB re 1 V/µPa, Loggerhead Instruments, FL, USA) was deployed on a sandy bottom (40 m depth) in front of STARESO, in Calvi, Corsica (France; 42.5801°N, 8.7285°E). The DSG recorded five minutes per hour at a sample rate of 20 kHz, from 7 June 2013 to 2 July 2013 (N_recordings_ = 613; 25 days on a 24-hour cycle).

#### Sound analysis

The first, third and fifth minutes of every hourly recording (five-minute samples) were visually and aurally inspected using Raven Pro 64 1.4 (Bioacoustic Research Program, Cornell Laboratory of Ornithology, Ithaca, NY, USA). Data were manually analysed for sound diversity (number of sound types) and sound abundance (abundance of each sound type). The measured sound types were S (=Sciaenidae sounds), O (=*O*. *rochei* sounds); *Kwa* sound (=harmonic sounds of unknown origin); PS (=pulse series sounds of unknown origin) and OP (=single pulse sounds of unknown origin). These sound types were labelled based on previously identified sound characteristics (e.g.^39,47–49^), while for fish sounds emitted by unknown species, a nomenclature frame developed by Di Iorio was adopted (e.g. “Kwa”^35^ and PS; Di Iorio,in review). Acoustic features of 20 sounds per sound type were assessed using Raven Pro 64 1.4. These included peak frequency (Peak freq, Hz), duration of the sound (DUR, s), pulse period (PP, s), and number of pulses (NP). Principal component analysis (PCA) carried out with Minitab 17, confirmed that this classification was based on mutually exclusive sound types (Supplementary Fig. 2 and Supplementary Table 7). The abundance of each sound type was measured on an ordinal scale, ranging from 0 (absence, i.e. no sound production) to 4 (chorus), following^50,51^) (Supplementary Table 8). The total sound abundance resulted from the sum of the abundance of all sound types.

#### Acoustic Complexity Index calculation and statistical analysis

Each five-minute recording (N = 613) was down-sampled to 8 kHz to calculate the ACI across the frequency range 0–4000 Hz, using a temporal resolution of 0.5 s, FFT = 512 (Hanning window; ∆fi 15.625 Hz) and by setting the amplitude filter to 0 μV^2^/Hz. However, to understand the influence of the noise filter, ACI computation was repeated, on both original tracks and on amplified tracks (+20 dB), by setting the filter at 2000 μV^2^/Hz and 5000 μV^2^/Hz, respectively. Finally, ACI values were summed for the frequency band 0–2000 Hz (ACIsum). Spearman rank correlation was used to investigate the strength of the relationship between number of sounds (i.e. sound abundance) and number of sound types (i.e. sound diversity) with the ACI.

## Electronic supplementary material

Supplementary Materials
