# Supplementary material for: Acoustic Complexity of vocal fish communities: a field and controlled validation
Source: Sci Rep. 2018 Jul 12;8:10559. doi: 10.1038/s41598-018-28771-6 (PMC6043554; doi:10.1038/s41598-018-28771-6)
Supplement: Supplementary file 1 — Supplementary Materials [file 41598_2018_28771_MOESM1_ESM.pdf]

Acoustic Complexity of vocal fish communities: a field and controlled validation.

Marta Bolgan, M. Clara P. Amorim, Paulo J. Fonseca, Lucia Di Iorio and Eric Parmentier

Supplementary Information

Supplementary Table 1. Controlled experiment: difference in the ACI values when changing frequency resolution or temporal resolution. Statistically significant differences (Bonferroni correction) are highlighted (asterisk).

	**Frequency resolution influence (Δf_i, Hz)**							**Temporal resolution influence (s)**			
	**H**<br>***p-value***	**15.6** *vs* **31.2**	**15.6** *vs* **7.8**	**15.6** *vs* **93.7**	**31.2** *vs* **7.8**	**31.2** *vs* **93.7**	**7.8** *vs* **93.7**	**H**<br>***p-value***	**0.5** *vs* **5**	**0.5** *vs* **10**	**5** *vs* **10**
		U<br>*p-value*							*U*<br>*p-value*		
**20 sounds per minute (total)**											
**1 sp.**	47.01<br>0.001*	16189<br>0.849	57459<br>0.005*	32367<br>0.810	29188<br>0.050	16372<br>0.901	58475<br>0.014	0.289<br>0.865	n.a.		
**2 sp.**	65.84<br>0.001*	15960<br>0.679	64249<br>0.657	22832<br>0.000*	32462<br>0.870	11818<br>0.000*	44900<br>0.000 *	8.19<br>0.016 *	31429<br>0.40	28419<br>0.001 *	29609<br>0.06
**3 sp.**	89.17<br>0.001*	15501<br>0.389	62206<br>0.250	20320<br>0.000*	32579<br>0.919	10433<br>0.000*	42517<br>0.000 *	10.05<br>0.005 *	32672<br>0.95	28440<br>0.001 *	27954<br>0.004*
**60 sounds per minute (total)**											
**1 sp.**	49.88<br>0.001*	16061<br>0.753	56902<br>0.002*	32615<br>0.927	29137<br>0.052	16149<br>0.819	57510<br>0.005 *	0.10<br>0.950	n.a.		
**2 sp.**	70.49<br>0.001*	15961<br>0.680	65085<br>0.876	23009<br>0.000*	31616<br>0.538	11812<br>0.000*	43734<br>0.000 *	13.5 *<br>0.001 *	29106<br>0.028	27443<br>0.001 *	29563<br>0.05
**3 sp.**	100.94<br>0.001*	15598<br>0.443	61296<br>0.143	19353<br>0.000*	32278<br>0.793	10100<br>0.000*	41254<br>0.000 *	23.5<br>0.000 *	28173<br>0.006 *	25556<br>0.000 *	28027<br>0.004*
**100 sounds per minute (total)**											
**1 sp.**	46.41<br>0.001*	16127<br>0.802	57415<br>0.005*	32494<br>0.870	29258<br>0.060	16272<br>0.913	58227<br>0.010	0.11<br>0.944	n.a.		
**2 sp.**	69.59<br>0.001*	15590<br>0.439	64599<br>0.746	22180<br>0.000*	31508<br>0.501	12142<br>0.000*	44587<br>0.000 *	19.9<br>0.000 *	28173<br>0.001 *	26410<br>0.000 *	30083<br>0.10
**3 sp.**	101.66<br>0.001*	15187<br>0.243	62395<br>0.278	19183<br>0.000*	31895<br>0.641	10410<br>0.000*	40979<br>0.000 *	32.6<br>0.000 *	25102<br>0.001 *	24483<br>0.000 *	31017<br>0.30

Supplementary Table 2. Controlled experiment: difference in the ACI when changing the amplitude filter (µV^2/Hz) on recordings with fish choruses (CE_2). Statistically significant differences (Bonferroni correction) are highlighted (asterisk).

	**Amplitude filter**		
	**0 *vs* 2000**	**0 *vs* 5000**	**2000 *vs* 5000**
**Chorus of *S. umbra alone***			
U<br>*p-value*	1170.0<br>0.001 *	122.0<br>0.001 *	2733.0<br>0.002 *
**Chorus of *S. umbra* with low abundance of *O. rochei* and of *Kwa* sounds**			
U<br>*p-value*	871.0<br>0.001 *	32910.0<br>0.001 *	20502.0<br>0.001 *
**Chorus of *S. umbra* with high abundance of *O. rochei* and of *Kwa* sounds**			
U<br>*p-value*	1503.0<br>0.001 *	32905.0<br>0.001 *	21871.0<br>0.001 *

Supplementary Table 3. Calvi (France) fish vocal community: Spearman rank correlation coefficients between the ACI (ACIsum) and fish sound measurements (i.e. sound abundance = number of sounds, and sound diversity = number of sound types) when changing the amplitude filter settings (µV^2/Hz) and the amplification of the acoustic tracks. Statistically significant differences (Bonferroni correction) are highlighted (asterisk).

**Track specification &<br>ACI noise filter settings**	**Fish sounds<br>(type of measurement)**	r_s	***p-value***
Original track<br>Amplitude filter not activated	Sound abundance	0.70	0.000 *
	Sound diversity	0.68	0.000 *
Original track<br>Amplitude filter 2000	Sound abundance	-0.25	0.000 *
	Sound diversity	-0.33	0.000 *
Original track<br>Amplitude filter 5000	Sound abundance	-0.25	0.000 *
	Sound diversity	-0.28	0.000 *
Amplified track (+20 dB)<br>Amplitude filter not activated	Sound abundance	0.65	0.000 *
	Sound diversity	0.64	0.000 *
Amplified track (+20 dB)<br>Amplitude filter 2000	Sound abundance	0.37	0.000 *
	Sound diversity	0.31	0.000 *
Amplified track (+20 dB)<br>Amplitude filter 5000	Sound abundance	-0.08	0.001 *
	Sound diversity	-0.13	0.041 *

Supplementary Table 4. Calvi (France) fish vocal community: Spearman rank correlation coefficients between the ACI (ACIsum), the number of sound types (sound diversity), the number of sounds (sound abundance), the relative abundance of each sound type, and the presence of boats. S = *Sciaenidae* sounds; O = *O. rochei* sounds (male); *Kwa* = harmonic sounds of unknown origin; PS = pulse series sounds of unknown origin; and OP = single pulse sounds of unknown origin.

	**SOUND DIVERSITY**	**SOUND ABUNDANCE**	**S**	**O**	***Kwa***	**PS**	**OP**	**BOAT**
r_s	**0.68**	**0.70**	**0.18**	**0.58**	**0.66**	**0.12**	**0.26**	**-0.65**
p-value	**0.000**	**0.000**	**0.000**	**0.000**	**0.000**	**0.000**	**0.000**	**0.000**

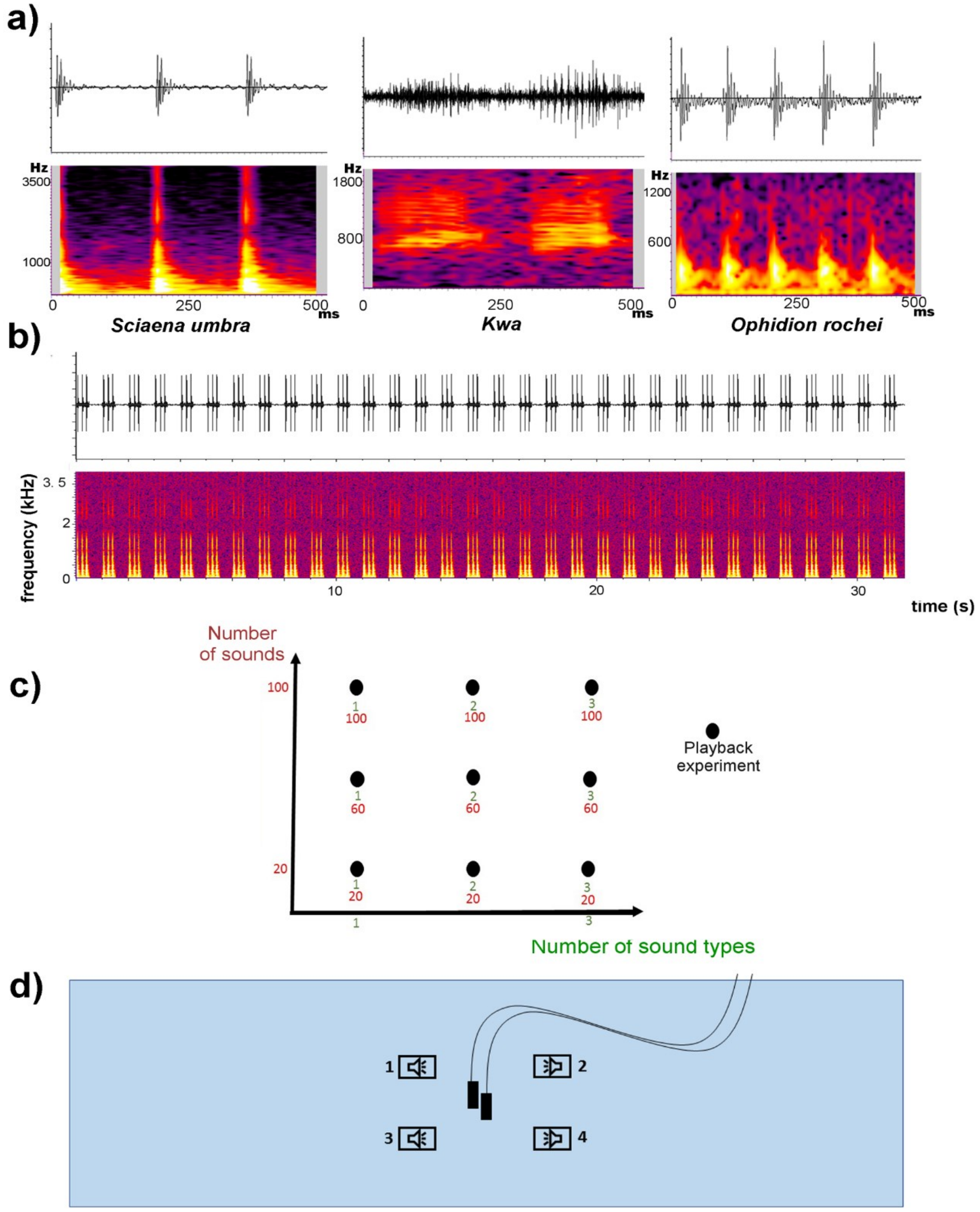

Supplementary Figure 1. The controlled experiment (CE): a) sound stimuli (500 ms); b) waveform and spectrogram (Hanning window, 512 FFT) of a portion (i.e. 30 s out of 3 min) of one of the tracks that were artificially created for the controlled experiment CE; c) design of CE_1 i.e. sound abundance (i.e. number of sounds) vs. sound diversity (i.e. number of sound types). Each black dot represents a playback experiment (and therefore one recording collected by the HTI hydrophone on which the ACI was subsequently calculated). The green numbers (upper) digits represent the number of sound types (i.e. sound diversity) that were presented in each playback, while the red number (lower) digits represent the number of sounds (i.e. sound abundance). (d) playback set-up.

Supplementary Table 5. The sound stimuli presentation during the controlled experiment (CE).

**CE_1 Sound abundance vs Sound diversity**			
**Loudspeaker 1**	**Loudspeaker 2 (*S.umbra*)**	**Loudspeaker 3 (*Kwa*)**	**Loudspeaker 4 (*O. rochei*)**
**TOTAL ABUNDANCE=20 sounds x min^{-1}**			
off	20	Off	Off
off	10	10	Off
off	7	7	6
**TOTAL ABUNDANCE=60 sounds x min^{-1}**			
off	60	Off	Off
off	30	30	Off
off	20	20	20
**TOTAL ABUNDANCE=100 sounds x min^{-1}**			
off	100	Off	Off
off	50	50	Off
off	33	33	33
**CE_2 The effect of fish chorus**			
off	Chorus	Off	Off
off	Chorus	20 sounds x min^{-1}	20 sounds x min^{-1}
off	Chorus	33 sounds x min^{-1}	33 sounds x min^{-1}
**CE_3 The effect of boat noise**			
off	50 sounds x min^{-1}	50 sounds x min^{-1}	Boat noise
off	30 sounds x min^{-1}	30 sounds x min^{-1}	Boat noise
off	20 sounds x min^{-1}	Off	Boat noise
**CE_4 The effect of rare sounds**			
off	2 sounds x min^{-1}	Off	Off
off	off	2 sounds x min^{-1}	Off
off	off	Off	2 sounds x min^{-1}
**CE_5 Sound abundance vs Sound diversity (with sounds at their real duration)**			
**ONE SOUND TYPE**			
off	Low abundance (25 sounds x min^{-1})	Off	Off
off	High abundance (50 sounds x min^{-1})	Off	Off
off	off	Low abundance (50 sounds x min^{-1})	Off
off	off	High abundance (100 sounds x min^{-1})	Off
off	off	Off	Low abundance (5 sounds x min^{-1})
off	off	Off	High abundance (10 sounds x min^{-1})
**TWO SOUND TYPES**			
off	Low abundance (25 sounds x min^{-1})	Low abundance (50 sounds x min^{-1})	Off
off	High abundance (50 sounds x min^{-1})	High abundance (100 sounds x min^{-1})	Off
**THREE SOUND TYPES**			
off	Low abundance (25 sounds x min^{-1})	Low abundance (50 sounds x min^{-1})	Low abundance (5 sounds x min^{-1})
off	High abundance (50 sounds x min^{-1})	High abundance (100 sounds x min^{-1})	High abundance (10 sounds x min^{-1})

Supplementary Table 6. Explanation of the terminology adopted in this manuscript, with reference to the ACI equation as taken from [18].

Term	Definition
Diversity; species diversity	Number of distinct species present in a given sampling area.
Sound diversity	Total number of sound types.
Sound abundance	Number of sounds. It can refer to the number of sounds of one single sound type (relative abundance), or it can refer to the sound abundance of all sound types (sound abundance).
Settings	The settings are the parameters that must be chosen by the operator in order to calculate the Acoustic Complexity Index. These are<br>1. Frequency resolution<br>2. Temporal resolution of the ACI algorithm<br>3. Amplitude filter
Frequency resolution (Δf_i)	Δf_i =sample rate/ FFT segment size
Temporal resolution	The temporal resolution (in seconds) at which the ACI algorithms are applied. See [45] for details, where the temporal resolution is called “clumping”, i.e. j.
Amplitude filter	This filter excludes all of the data that has an amplitude equal to, or less than, the selected value from the computation [45].<br>The filter cleans diffuse, feeble background noise (distributed in every frequency) from the sound file. The background noise largely depends on the environmental context in which the sound recorders operate. See [45] for details.
Acoustic Complexity Index (ACI)	Index developed and validated by Pieretti et al. [18] to infer the singing activity of an avian community.<br>Taken from [18]: On the basis of a matrix of intensities extrapolated from the spectrogram (divided into temporal steps and frequency bins Δf_i), the ACI calculates the absolute difference (dk) between two adjacent values of intensity (Ik and $I_{(k+1)}$) in a single frequency bin (Δf_i):<br>$dk = \|Ik - I_{(k+1)}\|$<br><br>and then adds together all of the dk encompassed in the first temporal step of the recording (j)<br><br>$D = \sum_{k+1}^{n} dk$ for $j = \sum_{k+1}^{n} \Delta t_k$<br>n= number of Δt_k in j<br>where D is the sum of all the d_k contained in j.<br><br>In order to obtain the relative intensity, and to reduce the effect of the distance of the birds from the recording microphone, this result is then divided by the total sum of the intensity values registered in j:<br><br>$ACI = \frac{D}{\sum_{k=1}^{n} I_k}$<br><br>where the ACI is calculated in a single temporal step (j) and in a single frequency bin (Δf_i). Thereafter, the ACI, which was worked out on all of the temporal steps

	encompassed in the recording, is calculated:<br>$$ACI_{\Delta fi} = \sum_{j=1}^{m} ACI$$<br>m= number of j in the entire recording<br><br>where the ACI(Δf_i) corresponds to the ACI of an entire frequency bin. Finally, the total ACI for all of the frequency bins is calculated:<br>$$ACI_{tot} = \sum_{l=1}^{q} ACI_{(\Delta fi)}$$<br>For<br>$$\Delta f = \sum_{l=1}^{q} \Delta fi_i$$<br>q= number of Δf_i
ACIsum	Sum of $ACI_{(tot)}$ in the frequency range 0-2000 Hz (i.e. frequency range in which fish vocalisations occur)

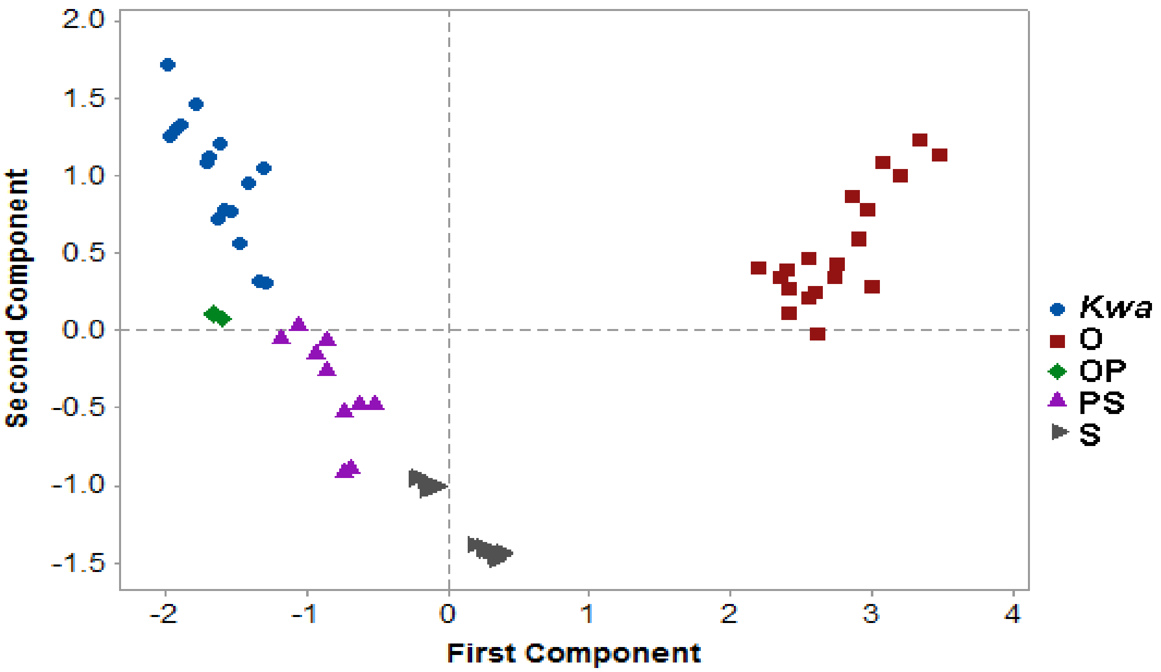

Supplementary Figure 2. Score plot of the Principal Component Analysis (PC1 and PC2) carried out on fish sounds recorded in Calvi (France). S = Sciaenidae sounds; O = O. rochei sounds (male); Kwa = harmonic sounds of unknown origin; PS = pulse series sounds of unknown origin; and OP = single pulse sounds of unknown origin.

Supplementary Table 7. Relevant coefficients of the Principal Component Analysis carried out on fish sounds recorded in Calvi (France). Peak freq = peak frequency; DUR = duration; PP = pulse period; NP = number of pulses.

Variables	PCA1	PCA2	PCA3	PCA4
Peak freq (Hz)	-0.444	0.668	-0.589	0.0980
DUR (s)	0.532	0.389	0.162	0.734
PP (s)	0.519	-0.343	-0.783	-0.022
NP	0.501	0.534	0.116	-0.671

Supplementary Table 8. Detailed explanation of the ordinal scale used to estimate the relative sound abundance of each sound type encountered in the recordings collected in the field (F). Sound abundance resulted from the sum of the relative abundance of all sound types.

Relative abundance	Sciaenidae sounds	*Ophidion rochei* and PS	*Kwa* sounds
0	No sound production	No sound production	No sound production
1	< 30 pulses $\times\ min^{-1}$	1 sound $\times\ min^{-1}$	Rare sounds (less than 10 $\times\ min^{-1}$)
2	30 < pulses < 50 $\times\ min^{-1}$	2 to 3 sounds $\times\ min^{-1}$	Some sounds (10 < sounds < 50 $\times\ min^{-1}$
3	> 50 pulses $\times\ min^{-1}$	> 3 sounds $\times\ min^{-1}$	Mass production of sounds in which the single sounds are still distinguishable.
4	Chorus (mass production of sounds)	Chorus (mass production of sounds)	Chorus (mass production of sounds)
